# Green Synthesis of Gold Nanoparticles and Their Cytotoxic Effect in Breast Cancer: An Experimental and Theoretical Study

**DOI:** 10.1021/acsabm.5c01172

**Published:** 2025-07-31

**Authors:** Fausto Díaz-Sánchez, Jesús A. Arzola-Flores, Maura Cárdenas-García, Miguel A. García-Castro, Juana D. Santamaría-Juárez

**Affiliations:** † 3972 Facultad de Ingeniería Química de la Benemérita Universidad Autónoma de Puebla, 18 Sur y Av. San Claudio, C.P. 72570 Puebla Pue, México; ‡ Laboratorio de Fisiología Celular, Facultad de Medicina de la Benemérita Universidad Autónoma de Puebla, C.P. 72570 Puebla Pue, México

**Keywords:** gold nanoparticles, electrolyte, bisphthalimide, breast cancer, XTT colorimetric

## Abstract

In this work, the synthesis of gold nanoparticles was studied using an electrolyte generated from a bisphthalimide. Both the nanoparticles and the electrolyte were tested to determine their cytotoxic effects on breast cancer cells of the MCF-7 and MDA-MB-231 lines. For this purpose, 6000 cells were seeded per well in a 96-well plate, and the XTT colorimetric assay was used. Additionally, AutoDock and GOLD were used to determine the structure-structure interactions during molecular docking.

## Graphical abstract

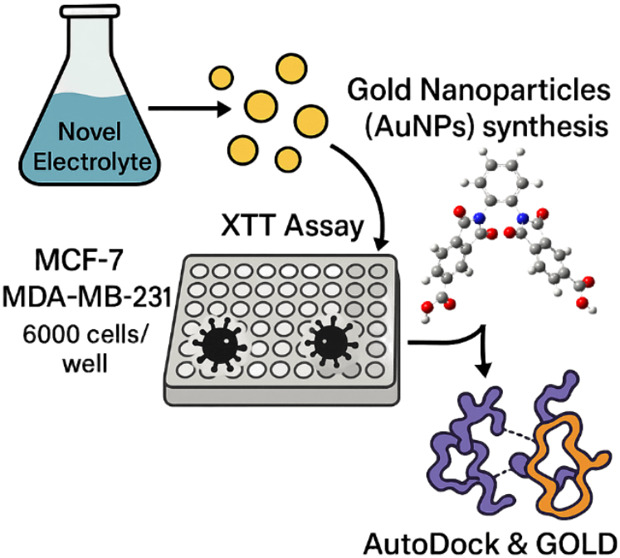

## 1 Introduction

Diimide-diacid and imide derivatives (DIDAs) have emerged as promising candidates in anticancer drug development due to their distinctive structural features and pharmacological potential.^1–6^ In particular, the ortho, meta, and para isomers (oDIDA, mDIDA, and pDIDA) represent a relevant class of molecules that have been successfully employed as electrolytes in the green synthesis of gold nanoparticles (AuNPs). This approach facilitates an efficient and environmentally sustainable process by eliminating the need for conventional reducing agents, which are often associated with toxicity and ecological concerns.^7–9^

Gold nanoparticles have attracted considerable attention in nanomedicine, particularly in oncology, owing to their unique physicochemical properties such as a high surface-area-to-volume ratio, tunable size, and surface functionalization capabilities. The combination of AuNPs with DIDAs, which integrate phthalimide moieties and carboxylic acid functional groups, enhances the biocompatibility and targeting efficiency, making them suitable for drug delivery and cancer therapy. Phthalimide-based compounds hold a well-established place in medicinal chemistry. Clinically relevant derivatives such as thalidomide and lenalidomide have demonstrated anticancer efficacy through mechanisms involving antiangiogenic, anti-inflammatory, and immunomodulatory effects.^10–13^ The addition of carboxylic acid groups improves solubility and strengthens interactions with biological targets, facilitating uptake and enhancing therapeutic potential.^14^

Breast cancer remains the most frequently diagnosed cancer and the leading cause of cancer-related death among women worldwide. Its incidence continues to rise, especially in low- and middle-income countries. Pathologically, breast cancer comprises a heterogeneous group of diseases classified into molecular subtypes (including luminal A, luminal B, HER2-enriched, and triple-negative), each with distinct prognoses and therapeutic responses. This complexity underscores the urgent need for innovative, selective, and less toxic treatment modalities. Recent studies indicate that AuNPs, especially when functionalized with bioactive ligands, can exert selective cytotoxic effects on cancer cells through mechanisms such as reactive oxygen species (ROS) generation, mitochondrial dysfunction, and apoptosis induction.^15–19^ In this context, preliminary evidence suggests that DIDAs can trigger apoptosis via the mitochondrial pathway, as indicated by increased caspase-3 activity and DNA fragmentation.^20,21^ Their carboxylic groups likely enhance interactions with key proteins involved in cell signaling, proliferation, and survival.

Structure–activity relationship (SAR) analyses reveal that the position of the phthalimide units significantly influences the anticancer activity. Among the isomers, pDIDA often exhibits superior cytotoxicity, likely due to its optimal spatial orientation for target interaction.^22–24^ Furthermore, the aromatic and planar nature of these compounds suggests potential for DNA intercalation, disrupting replication and transcription and contributing to their antiproliferative effects.^25^ Of particular interest is the observed selective cytotoxicity of certain DIDA derivatives, which demonstrate enhanced activity against cancer cells while sparing normal cells. This selectivity may arise from differences in membrane composition, metabolic profiles, or expression levels of target receptors between healthy and malignant cells.^26,27^ Additionally, the thermal stability, structural modifiability, and scalability of DIDAs make them attractive for pharmaceutical development. A recently developed solid-state synthesis for oDIDA provides an eco-friendly, high-yield route to these compounds, further supporting their applicability in large-scale production.^28,29^

In conclusion, DIDAs represent a novel and promising class of compounds for anticancer therapy, combining the benefits of green chemistry, targeted nanomedicine, and multifunctional pharmacophores. Further in-depth studies are needed to fully elucidate their mechanisms of action, refine SAR insights, and evaluate the in vivo efficacy in relevant cancer models.

## 2 Materials and Methods

### 2.1 Synthesis of Gold Nanoparticles (AuNPs)

Gold nanoparticles were synthesized by the colloidal method using N,N′-(1,2-phenylene)­bis­(phthalimide-5-carboxylic acid) (oDIDA) as a reducing and stabilizing agent. oDIDA was synthesized by solid-state reaction without a catalyst and without the use of condensing agents,^30^ then the compound was neutralized with NaOH in stoichiometric quantities, Figure 1. Solutions of oDIDA and HAuCl_4_·3H_2_O (1 × 10^–3^ M) were prepared in ultrahigh-purity water (ρ = 18 MΩ). 5 mL portion of oDIDA solution was mixed with 1 mL of HAuCl_4_·3H_2_O solution in a glass vial at room temperature. No additional reducing or stabilizing agents were used. Mixtures were protected from the light and remained at rest at room temperature. The evolution of the colloidal solutions was monitored by UV–Vis spectroscopy at different time intervals. The size and shape of the nanoparticles were analyzed by DLS (Dynamic Light Scattering) using a Malvern Zetasizer Nano Series ZS90. All the measurements were made in triplicate.

**Figure 1:**
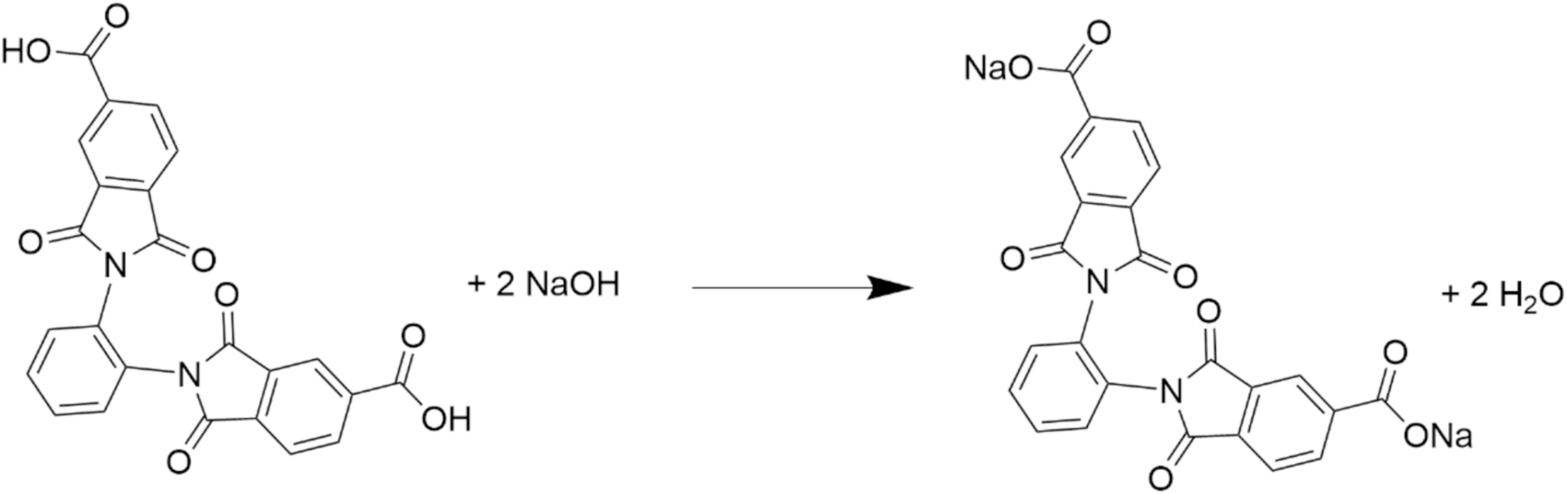
Neutralization of oDIDA.

### 2.2 Cell Culture

MDA-MB-231 cells were cultured in Dulbecco’s modified Eagle’s medium (DMEM) (Sigma-Aldrich), and MCF-7 cells were cultured in minimal essential medium (MEM) (Sigma-Aldrich).

Cell lines were supplemented with 7 and 10% fetal bovine serum (Biowest), 2% antifungal antibiotic (Sigma-Aldrich), and 0.1% l-glutamine (Sigma-Aldrich). They were then incubated at 37 °C in a humidified atmosphere with 5% CO_2_, according to Segeritz and Vallier.^31^ Cells were monitored every 24 h until reaching 80% confluence.

Once 80% confluence was reached, the adhesion of the cell cultures was inhibited by 1× trypsin, and they were placed in the incubator for 5 min; then the cultures were observed under the inverted microscope to confirm their separation. The next step was to inhibit the trypsin using twice the supplemented culture medium. For this point, a specific medium was used for this process; this medium contains only 2% fetal bovine serum (FBS). Once the trypsin was inhibited, the contents were emptied into a 15 mL Falcon tube and centrifuged at 1500 rpm for 10 min; once this process was complete, a pellet containing all the cells was observed in the Falcon tube, the supernatant was removed, and the pellet was resuspended with fresh culture medium.

Next, 45 μL of phosphate-buffered saline (PBS), 45 μL of trypan blue, and 10 μL of the pellet solution with medium (this solution was homogenized prior to incorporation) were added to an Eppendorf tube. Finally, 10 μL of the previous solution was added to a 0.1 mm Neubauer chamber, and this solution was observed under the microscope for cell counting.

#### Cytotoxic Effect

From each line, 6000 cells per well were seeded in 96-well plates.^32^ The volume was then supplemented with fresh culture medium to 200 μL per well, and the cells were incubated for 24 h. The culture medium was then removed, each well was washed with PBS (to remove cell debris), and macromolecules were added at concentrations of 100, 75, 50, 25, and 10 μM, as well as a negative control in which cells were seeded, and no treatment was added.

The antiproliferative effect was determined by using the XTT cell proliferation assay kit (5 mL of proliferation vial and 100 μL of activator) by adding 50 μL of reagent per well and incubating again for 4 h. Finally, the absorbance was measured at 450 nm using a Multiskan FC microplate photometer 51119000 with a wavelength range of 340 to 850 nm and ± 1% (0 to 3 Abs) or ± 0.003 Abs (the highest value at 405 nm).^33^ All experiments were performed in triplicate.

#### Noncancerous Cell Control

The normal breast epithelial line MCF-10A was used as a noncancerous cell model to determine the toxicity of the macromolecules; these cells were maintained in DMEM/F12 culture (Sigma-Aldrich) and supplemented with 7% fetal bovine serum (Biowest), 2% antifungal antibiotic (Sigma-Aldrich), 0.1% l-glutamine (Sigma-Aldrich), and 1 μg/mL hydrocortisone (Sigma-Aldrich) and incubated at 37 °C in a humid atmosphere with 5% CO_2_ monitored every 24 h until 80% confluence was reached.

The median inhibitory concentrations (IC_50_) were used.^34^ This is the concentration of a drug required to inhibit 50% of a biological activity in a given system; a low IC_50_ indicates a higher potency of a drug, so that less of the compound is required to produce the inhibitory effect, while a high IC_50_ indicates a lower potency of a drug, so that a higher amount is required to produce the same effect, the IC_50_ depends on the organic compound used. The IC_50_ values found in the MDA-MB-231 and MCF-7 lines were determined, with each concentration tested three times and in triplicate. Finally, the XTT assay was used to measure the antiproliferative effect.

### 2.3 Molecular Docking

The *SwissTargetPrediction* program was used to determine the probability of interaction of all oDIDA with the proteins of the different signaling pathways identified for breast cancer.^35,36^ The main criterion for selecting a protein from the Protein Data Bank (PDB) was that it had been analyzed by XRD and that it had a resolution of ≤ 2.0 Å. In addition, the three-dimensional minimum energy solution for the compound studied was obtained using the Avogadro software and the SMILES code, using molecular mechanics, the MMFF94 level of theory.^37^

Molecular docking results were obtained using AutoDock 4.2.6, and finally, using GOLD.^38,39^ The compound was compared for each molecule with inhibitors found in the literature to see if oDIDA could replace them.

### 2.4 Tauc′s Theory

Accurate determination of the band gap (*E*_g_) in nanoscale materials is essential to understanding their electronic and optical properties.

A widely used tool for this purpose is the Tauc theory, which allows estimation of the *E*_g_ value from UV–vis spectroscopy data. Although originally developed for amorphous semiconductors, this approach has been extensively applied to nanomaterials, including metallic ones, such as gold nanoparticles (AuNPs), particularly when their size is reduced to below 5 nm.^40^

In this nanoscale regime, AuNPs undergo marked quantum confinement, leading to the appearance of an optical pseudogap and a substantial modification in their band structure. As the particle size decreases, the electronic levels become discretized and the density of states moves away from that typical of bulk metals, allowing the appearance of optical transitions that can be modeled by the Tauc eq (Ec. [Disp-formula eq1]).^41^

1 $${\left(\alpha h\nu \right)}^{1/n}=A\left(h\nu -E_{g}\right)$$

where α is the absorption coefficient, being a function of wavelength α­(λ), *h* is the Planck constant, *E*_g_ is the optical band gap of a semiconductor, ν is the frequency, *A* is a proportionality constant, and *n* is the Tauc exponent.

Conditions under which Tauc’s Method is Applicable for Gold Nanoparticles

1. Very small nanoparticles (<5 nm) and quantum confinement effect
  a. When gold nanoparticles are smaller than 5 nm, free electrons start to experience quantum confinement, which can induce a discrete separation of electronic levels.
  b. In this case, a semiconductor-like behavior appears, allowing the use of Tauc’s method to estimate an effective gap. For sizes in the 1–3 nm range, this effect is more pronounced, and *E*_g_ can vary between 1.5 and 2.5 eV depending on the size.

2. Gold nanoclusters with fewer than 100 atoms (size <2 nm) show a separation of electronic levels due to the transition from metallic to nonmetallic state.
  a. These systems can show an energy gap that varies with cluster size, allowing the Tauc method to be applied to analyze their optical behavior.
3 If the gold nanoparticles are oxidized or functionalized with molecules that modify their surface, the system may acquire different optical characteristics, which may lead to the appearance of an effective gap in its absorption spectrum.
  a. In these cases, Tauc’s method can be applied to estimate the optical transition energy of the modified system.

## 3 Results and Discussion

### 3.1 Synthesis and Characterization of AuNPs

One of the advantages of the colloidal method is that the shape and size of the nanostructures can be controlled by changing some variables, such as the stabilizer, concentration, and type of solvent. AuNPs were synthesized using oDIDA as both reducing and stabilizing agent, following the methodology described in previous studies.^7–9^ The first evidence of AuNPs formation was the appearance of a red color in the colloidal solution. The UV–Vis absorption spectrum recorded 8 min after the reaction began (Figure 2) displayed a characteristic band at 520 nm, corresponding to the localized surface plasmon resonance (LSPR) of AuNPs, similar to the spectra observed for their structural isomers. Regarding the temporal evolution, the color change in the oDIDA solution occurred significantly faster than in the mDIDA and pDIDA analogues, which required approximately 18 days and 24 h, respectively, to show a comparable response.^8^

**Figure 2:**
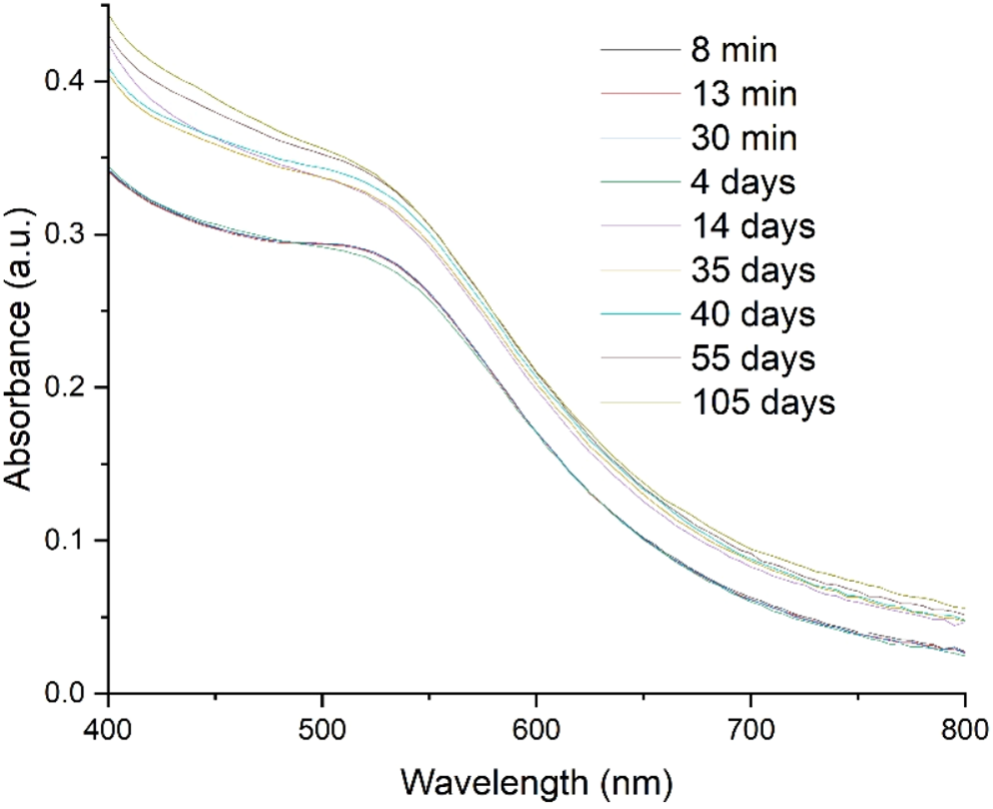
Temporal evolution of the UV–vis absorption spectra of a colloidal solution of AuNPs.

The electrostatic potential of the AuNPs was determined from the Zeta potential; this test was carried out in triplicate after 24 h of synthesis and after 105 days (Table 1), and these values indicate that the trend of stability tends to decrease. The results of the zeta potential counts in millivolts are shown in Figure 3.

**Figure 3:**
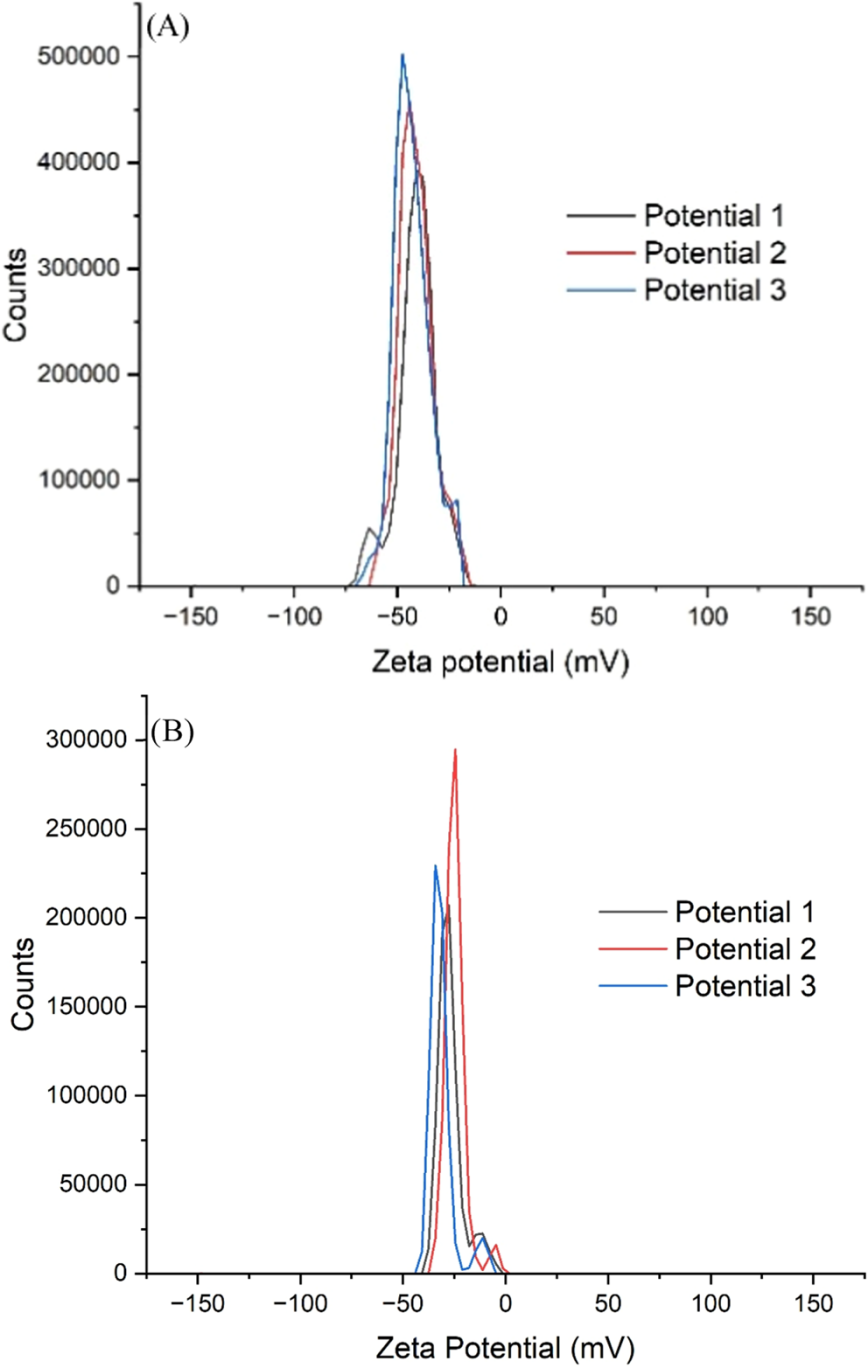
Zeta potential for oDIDA: (A) 24 h, (B) 105 days.

**Table 1:** Zeta Potential Obtained for oDIDA in mV

time	potential 1	potential 2	potential 3	average^a^
24 h	–41.0	–45.4	–45.1	–45.2 ± 2.5
105 days	–26.3	–26.8	–28.0	–27.0 ± 0.9

a Uncertainties represent the mean standard deviation.

Figure 4 shows that although the compound did not produce monodisperse nanoparticles, the average particle size obtained from three independent experiments with oDIDA was (5.0 ± 2.5) nm, where the uncertainty represents the average standard geometric deviation. According to this technique, the morphology of the AuNPs was predominantly spherical and/or quasi-spherical.^40,41^ As observed, the size, shape, and low polydispersity of the nanoparticles synthesized with oDIDA are consistent with previously reported results for both mDIDA and pDIDA-based systems.^8^

**Figure 4:**
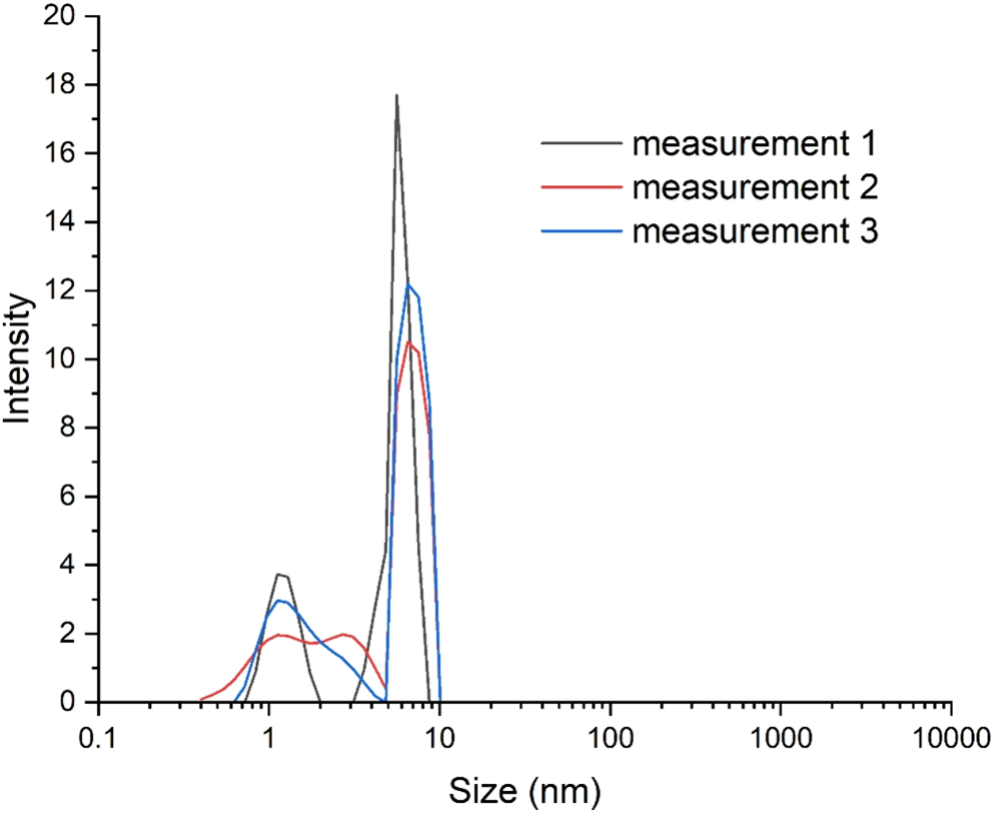
Size of AuNPs synthesized by oDIDA.

### 3.2 Theoretical Methods

#### 3.2.1 Tauc Equation

A value of *n* = 1/2 was used for AuNPs, which corresponds to direct transitions allowed for this material, and the value of the band gap energy (*E*_g_) was obtained for different times using the experimental values shown in Figure 2.

It was found that for a time of 8 min, an *E*_g_ of 1.97 eV was obtained, and for a total of 105 days, the *E*_g_ corresponds to 1.92 eV. The decrease in the “forbidden band” energy is due to the growth of the AuNPs since, with respect to the Zeta potential for this time, the particles were no longer stable. The particle size was calculated from the following expression.^42^

2 $$E_{g}\left(R\right)=E_{g,\mathrm{bulk}}+\frac{A}{R^{n}}$$

where *E*_g_ corresponds to the value obtained by the Tauc equation and, *E*_g,bulk_ corresponds to the value of the band gap for the material at the macroscopic level. In the case of gold, as it does not have a band gap, this term takes the value of 0, *A* is an empirical constant that takes values from 4.5 to 6.0 nm, *n* takes values from 1.3 to 1.5, and *R* is the radius of the nanoparticle in nm.^43^ Clearing eq 2 gives.

3 $$R={\left(\frac{A}{E_{g}}\right)}^{1/n}$$

Given the values, it was found that at 8 min the radius was 1.94 nm, and at 105 days it was 1.98 nm, corresponding to diameter values of 3.89 and 3.96 nm, respectively (see Figures S1–S3). These values are consistent with those obtained experimentally using DLS (5.0 ± 2.5) nm.^44^

It is important to highlight that although the Tauc method was originally developed for semiconductors, its application to AuNPs smaller than 5 nm has proven to be viable. This is primarily due to the pronounced quantum confinement effect observed at this scale, which leads to a noticeable separation between the HOMO and LUMO energy levels, effectively introducing an optical “band gap” in these metallic nanostructures. In such small dimensions, electrons are confined to such an extent that their energy levels become discretized, resulting in quantization effects similar to those observed in nanoscale semiconductors. Consequently, as the particle size decreases, the band gap widens (an established trend in quantum-confined systems). Although gold is a metal, ultrasmall AuNPs exhibit optical behavior that partially mimics that of semiconductors, particularly in how their absorption spectra shift with size due to changes in their discrete electronic states. In this study, the measured hydrodynamic diameters confirm that the nanoparticles fall within the size range where these quantum effects are expected to occur, thus supporting the applicability of the Tauc method. Therefore, for ultrasmall AuNP systems, the Tauc approach provides a valid and consistent method to estimate their optical transition energy.

#### 3.2.2 Molecular Docking

Table 2 shows the interaction probability for oDIDA with different proteins. In Table S1, the ID of each protein in the PDB is shown.

**Table 2:** Probability of Interaction of the oDIDA

protein	probability (%)	protein	probability (%)	protein	probability (%)
IGF1R^a^	0.1202	PARP1^d^	0.1202	FGF1^g^	0.1202
PI3Kγ^b^	0.1202	ESR2^e^	0.1202	GSK3α^h^	0.1202
MAPK1^c^	0.1202	APEX1^f^	0.1202	CDK2^i^	0.1202

a Taken from reference (^45^).

b Taken from reference (^46^).

c Taken from reference (^47^).

d Taken from reference (^48^).

e Taken from reference (^49^).

f Taken from reference (^50^).

g Taken from reference (^51^).

h Taken from reference (^52^).

i Taken from reference (^53^).

Based on the results obtained in Table 2, the couplings were carried out using AutoDock 4.2.6 software, the parameters used were a total of 100 runs per macromolecule, a number of energy evaluations of 2,500,000 (average type). For the calculations, the Lamarckian genetic algorithm was used, which has been shown to be the best for carrying out this type of study.^54^ The hydrogen bonds and other interactions generated during docking, as well as the binding distance, were visualized with the Biovia Discovery Studio software (Table S2).

Table 3 presents the R-L complexes obtained, and the best couplings were with GSK3α, MAPK1, and CDK2 proteins (Figures 5 and 6), based on the binding free energy, although CDK2 formed only two hydrogen bonds, which, according to the literature, are strong bonds due to their distance; the other hand, the complex generated with ESR2 and FGF1 formed a total of 8 hydrogen bonds, but between these two complexes, the best one turns out to be ESR2 (Figure 7), since the binding energy presents a more negative value and contains a greater amount of strong bonds, given the distance found, it is worth highlighting that during the couplings no unfavorable interaction was found in the R-L complexes.

**Figure 5:**
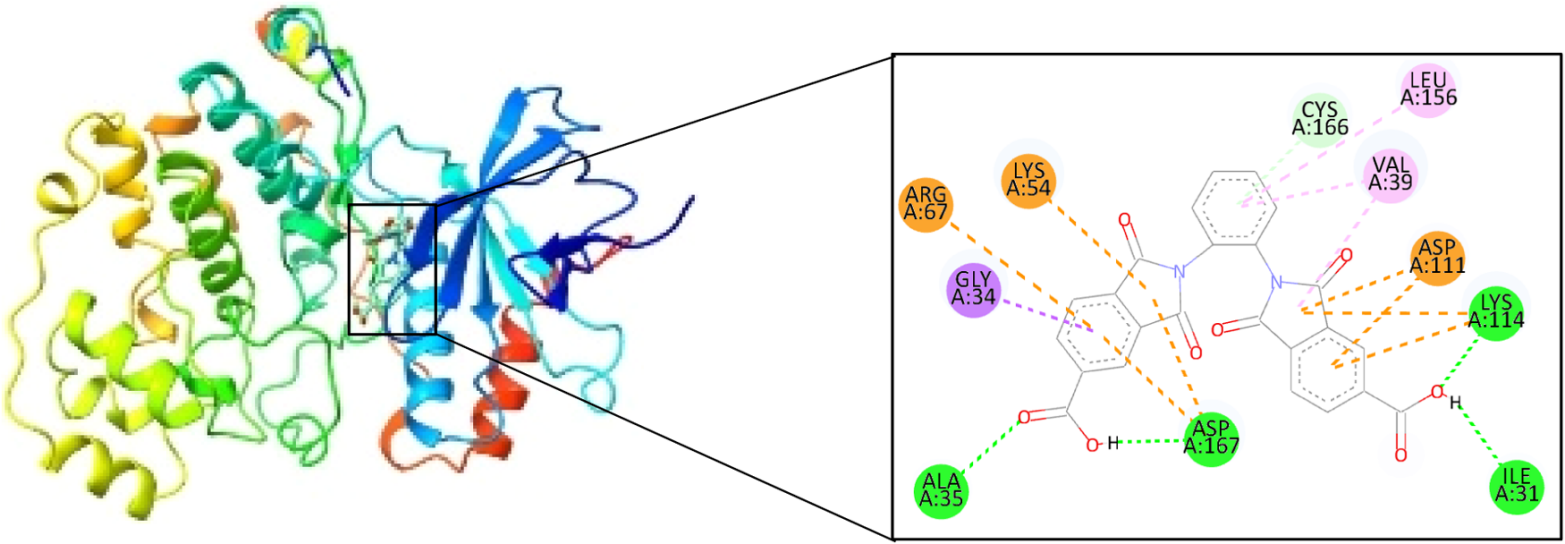
oDIDA binding site on the MAPK1 protein.

**Figure 6:**
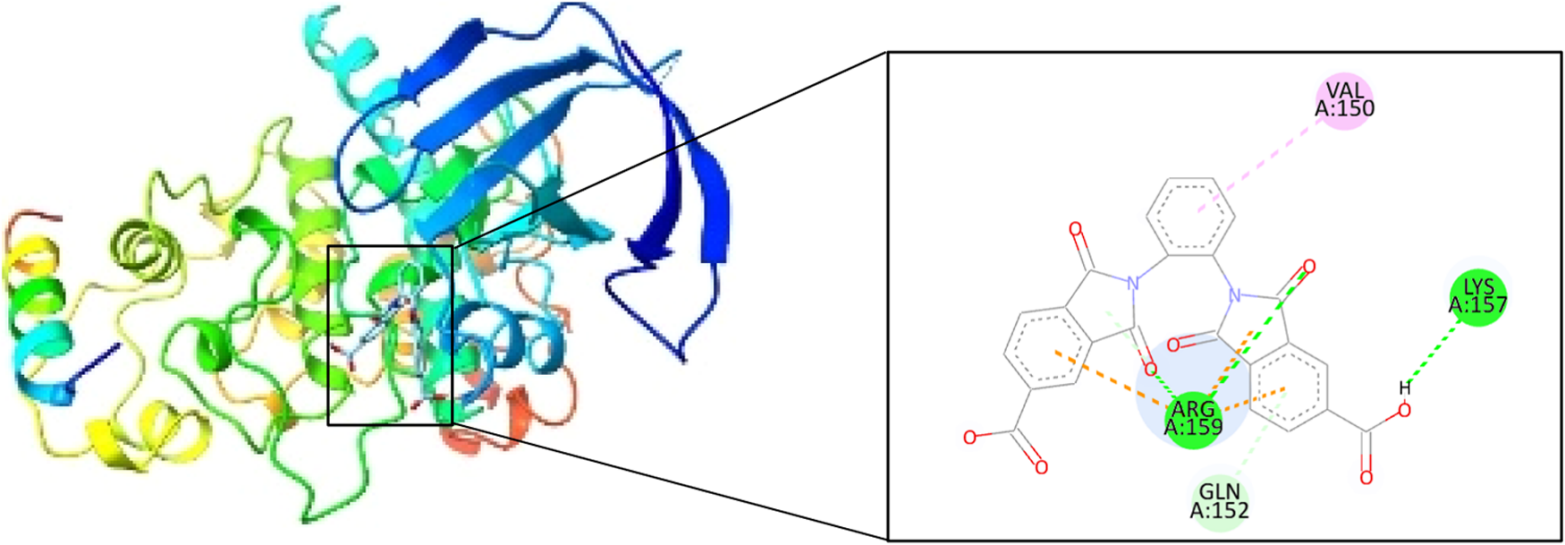
oDIDA binding site on GSK3α protein.

**Figure 7:**
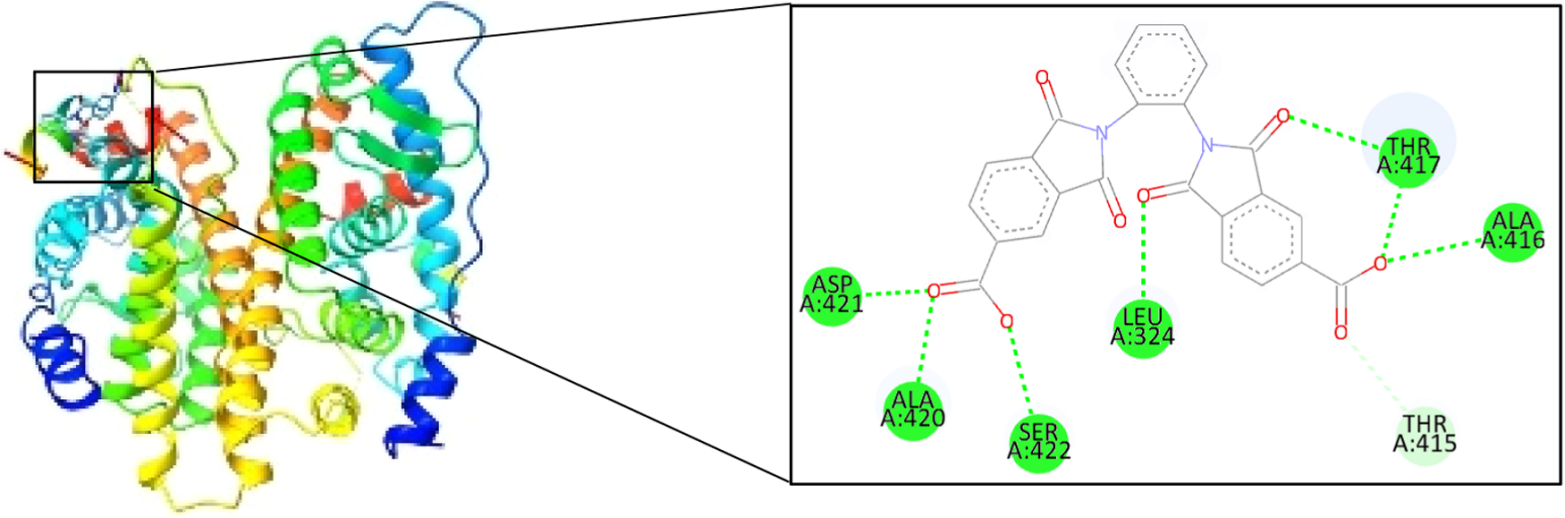
oDIDA binding site on ESR2 protein.

**Table 3:** Gibbs Binding Energies and Interactions with the Amino Acids of the Different Proteins Obtained from Molecular Docking

		interaction type^b^		
protein	–Δ_bind_ *G* ^a^	hydrogen bond	hydrophobic	electrostatic
PARP1	5	LYS945 (1.77)	TYR930 (5.55)	HYS934 (4.16)
		LYS945 (2.99)	LYS933 (4.74)	
		GLU931 (1.81)	LEU932 (5.16)	
		SER936 (2.47)	LEU932 (3.77)	
		LYS933 (1.96)	HIS934 (5.17)	
PI3Kγ	5.82	GLU546 (2.14)	TYR580 (5.12)	ARG579 (3.92)
		ARG579 (1.70)	TYR580 (4.53)	
		ARG613 (3.78)	PHE578 (5.76)	
ESR2	7.51	SER422 (2.24)	-	-
		ASP421 (1.77)		
		ALA420 (2.27)		
		LEU324 (1.91)		
		THR417 (2.68)		
		ALA416 (2.38)		
		THR417 (1.71)		
		THR415 (2.98)		
FGF1	6.36	HIS717 (2.32)	ARG661 (4.84)	
		SER699 (2.23)		
		ASN659 (2.37)		
		GLN574 (2.20)		
		ARG661 (2.29)	ARG661 (5.08)	
		LYS665 (2.63)		
		LYS665 (1.84)		
		PRO702 (3.02)		
IGF1R	4.71	LYS1171 (2.79)	GLY1172 (4.15)	CYS1171 (4.85)
		LYS1171 (2.34)	LEU1173 (5.36)	
		LEU1173 (1.76)	VAL1176 (4.57)	
		VAL1176 (2.27)	VAL1176 (5.09)	
		LEU1174 (2.44)		
GSK3α	10.59	LYS157 (2.06)	VAL150 (4.38)	ARG159 (4.93)
		ARG159 (2.77)		ARG159 (3.95)
		ARG159 (2.8)		ARG159 (4.13)
		ARG159 (2.19)		
		GLN152 (2.75)		
MAPK1	10.38	ALA35 (2.42)	GLY34 (3.45)	LYS54 (4.45)
		ASP167 (2.16)		ARG67 (4.93)
		ILE31 (2.56)	LEU156 (5.07)	ASP167 (4.21)
				ASP167 (3.8)
		LYS114 (1.83)	VAL39 (5.21)	ASP111 (4.04)
				ASP111 (3.79)
		CYS166 (3.93)	VAL39 (4.62)	LYS114 (4.88)
				LYS114 (4.32)
APEX1	9.76	SER100 (2.2)	LYS77 (4.76)	CYS99 (4.82)
		LYS98 (2.03)	ALA74 (5.09)	
		ALA74 (1.9)	ALA74 (4.53)	
			ALA74 (3.98)	
		ARG73 (2.75)	ARG73 (3.95)	
		ARG73 (2.38)	ARG73 (3.92)	
		ARG73 (3.33)	ARG73 (4.42)	
			ARG73 (3.82)	
CDK2	10.03	ARG200 (2.09)	LEU202 (3.93)	ARG200 (3.89)
			VAL251 (5.16)	
			PRO204 (4.65)	
		ARG200 (1.92)	PRO204 (4.03)	
			ARG200 (5.32)	
			ARG200 (3.95)	
			ARG200 (3.92)	

a The results are shown in kcal mol^–1^.

b Distances are in Å.

Table 4 summarizes the molecular docking scores obtained for the oDIDA compound in comparison with known inhibitors of the respective target proteins, as reported in the literature.^55–61^ The docking simulations were conducted using GOLD software, which evaluates ligand–protein binding based on genetic algorithms and scoring functions that estimate binding affinity. The results indicate that oDIDA exhibited a higher binding affinity, reflected in superior docking scores, for four key proteins: PI3Kγ, IGF1R, CDK2, and MAPK1. This suggests that oDIDA may effectively compete with or potentially replace the currently reported inhibitors for these molecular targets. Such findings highlight the compound’s potential as a multitarget anticancer agent. Furthermore, Figure 8 provides a visual representation of the molecular interaction between oDIDA and the active (inhibitor) site of the protein, illustrating its orientation, binding pose, and key interactions such as hydrogen bonds, hydrophobic, and electrostatic contacts.

**Figure 8:**
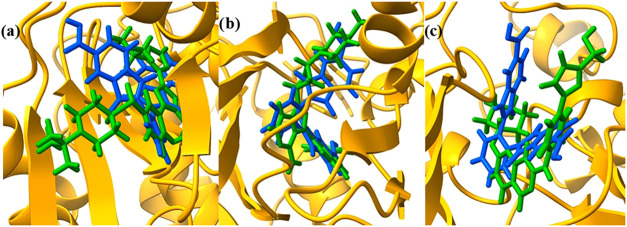
Comparison of the binding sites between oDIDA (blue) and the inhibitors (green): (a) PI3Kγ, (b) IGF1R, and (c) CDK2.

**Table 4:** Comparison of oDIDA and Inhibitors^a^

			amino acids					
protein	molecule	S^b^	GLN 117	PRO 105	TRP 119	HIS 116	LEU 114	PRO 112
APEX1	Lucigenin^c^	6	CH	VDW	VDW	VDW	VDW	VDW
	oDIDA		HB	VDW	VDW	HB	VDW	VDW
			ALA 144	VAL 18	LEU 134	ILE 10	LYS 129	GLY 13
CDK2	Roscovitine^d^	10	PA	PA	A	PA	VDW	VDW
	oDIDA		PA	PA	PA	PA	VDW	VDW
			KCX 33	PHE 80	VAL 64	HIS 84		
	Roscovitine		VDW	VDW	VDW	VDW		
	oDIDA		VDW	VDW	VDW	HB		
			LEU 188	GLU 137	TYR 134	CYS 199	VAL 70	ARG 141
GSK3α	AR-A014418^e^	6	PA	VDW	VDW	VDW	VDW	VDW
	oDIDA		PA	VDW	VDW	VDW	PA	HB
			LYS 1003	VAL 983	ALA 1001	LEU 975	VAL 1033	MET 1049
IGF1R	PQIP^f^	14	HB	PA	PA	PA	PA	PSU
	oDIDA		HB	PA	PA	PA	PA	PSU
			MET 1024	ASP 1123	PHE 1124	GLY 1125	ARG 1109	GLY 978
	PQIP		VDW	VDW	VDW	VDW	VDW	VDW
	oDIDA		VDW	VDW	VDW	VDW	VDW	VDW
			GLY 976	LEU 1051				
	PQIP		VDW	VDW				
	oDIDA		VDW	VDW				
			MET 125	ILE 48	LEU 173	TYR 53	LEU 124	GLN 122
MAPK1	SCH772984^g^	8	HB	PA	PA	PS	VDW	VDW
	oDIDA		HB	PA	PA	PP	VDW	VDW
			GLU 88	CYS 183				
	SCH772984		VDW	VDW				
	oDIDA		VDW	PA				
			HIS 862	TYR 896	HIS 862	LEU 877	TYR 889	SER 864
PARP1	WT^h^	6	CH	PP	PP	VDW	VDW	VDW
	oDIDA		CH	PP	PP	PA	VDW	VDW
			ASP 964	ILE 881	LYS 833	TYR 867	ILE 831	ILE 879
PI3Kγ	GNE-293^i^	17	HB	CH	PA	PP	PA	PA
	oDIDA		HB	PA	HB	HB	PA	PA
			ILE 963	MET 953	PRO 810	SER 806	ASP 841	LEU 838
	GNE-293		A/PA	PSU	VDW	VDW	VDW	VDW
	oDIDA		A/PA	PSU	VDW	VDW	HB	VDW
			PHE 961	ALA 885	THR 886	THR 887	MET 804	
	GNE-293		VDW	VDW	VDW	VDW	VDW	
	oDIDA		VDW	PA	VDW	VDW	PSU/PA	

a CH: carbon–hydrogen bond, HB: hydrogen bond, VDW: van der Waals, PA: Pi-alkyl interactions, PS: Pi-sigma interactions, PP: Pi-Pi interactions, A: alkyl interactions, PSU: Pi-sulfur interactions.

b Similarity.

c Taken from reference (^55^).

d Taken from reference (^56^).

e Taken from reference (^57^).

f Taken from reference (^58^).

g Taken from reference (^59^).

h Taken from reference (^60^).

i Taken from reference (^61^).

### 3.3 Cell Viability

MDA-MB-231 and MCF-7 cell lines were used, grown in T25 boxes to 80% confluence, then trypsinized and transferred to T75 boxes to 80% confluence, after which the cells were detached, centrifuged, and counted to determine the number of cells per μL, seeded at 6000 cells per well in a 96-well box.

After seeding the cells in the 96-well box, the culture medium was removed, the compound to be tested and the generated AuNPs were added in decreasing concentrations (100–10 μM) and in triplicate. The volume was supplemented with medium to 200 μL and incubated for 24 h. Finally, cell viability was measured using the XTT cell proliferation kit; as a negative control, cells were seeded without treatment (Figure 9).

**Figure 9:**
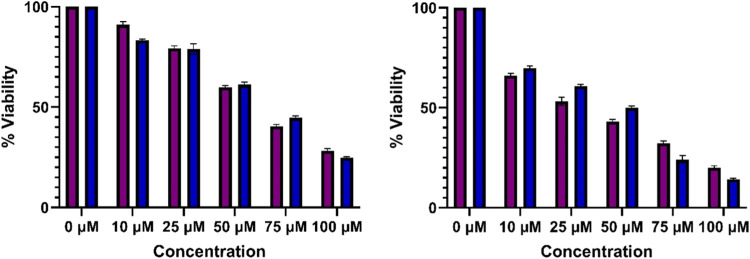
Cell viability (purple MDA-MB-231 and blue MCF-7) for oDIDA (left) and AuNPs obtained by oDIDA (right).

As can be seen, the boxplot shown in Figure 10 illustrates the differences in cell viability for each treatment condition, both for oDIDA alone and for AuNPs synthesized using oDIDA as a reducing and stabilizing agent. The figure shows a decrease in cell viability for both cases for the MDA-MB-231 cell line, with a more pronounced reduction observed for the AuNPs. To determine whether these differences were statistically significant, an ANOVA test was conducted after confirming the assumptions of normality and homoscedasticity using the Shapiro-Wilk and Levene’s tests, respectively.

**Figure 10:**
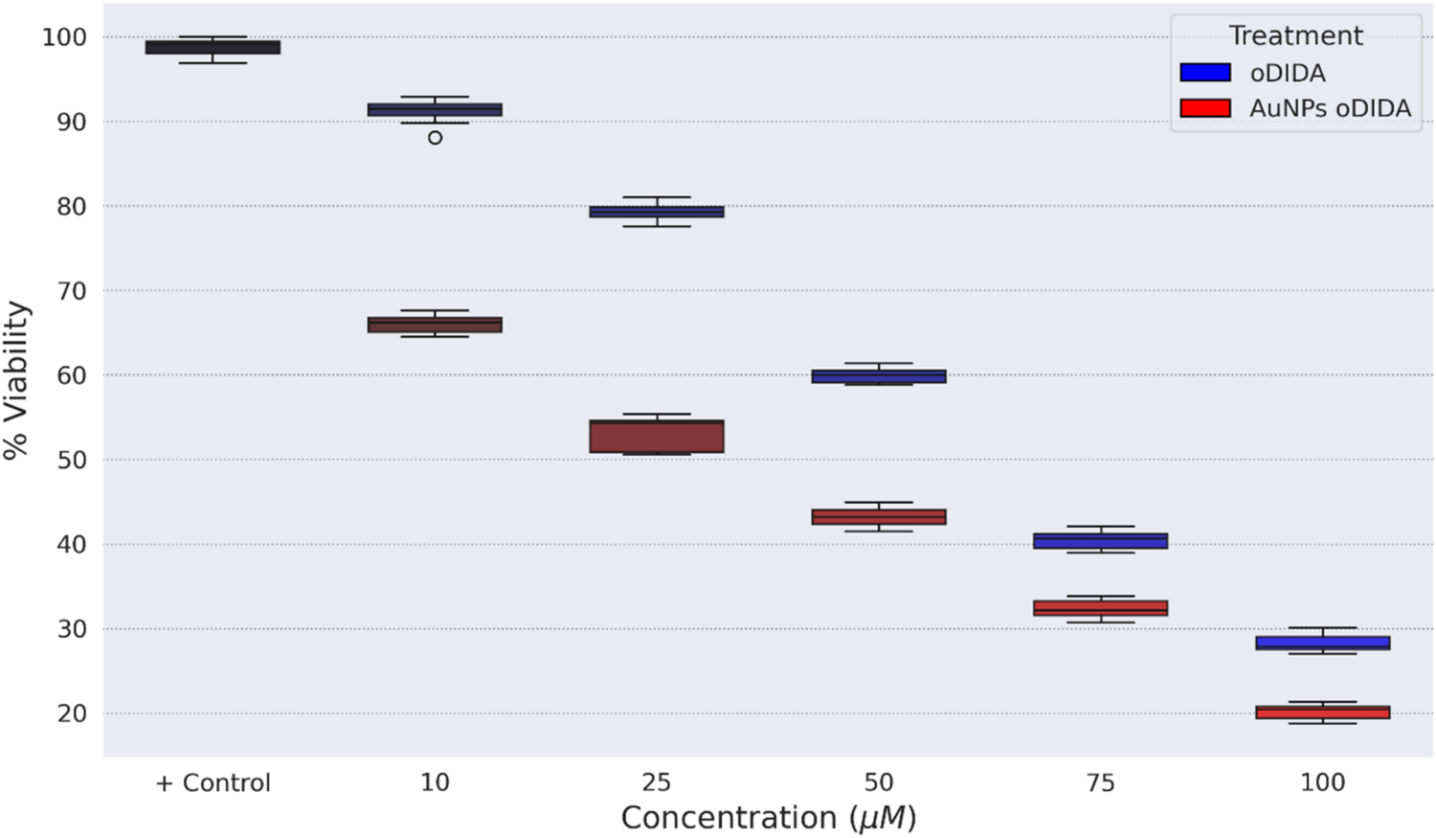
Boxplot of the cell viability percentage of the MDA-MB-231 cell line for each treatment.

Cell viability percentage was used as the response variable, while oDIDA and AuNPs concentrations were treated as factors with four treatment levels (25, 50, 75, and 100 μM). Post hoc Dunnett and Tukey HSD tests were performed to identify significant differences between the treatment groups and the control (Dunnett), as well as among the different treatment levels (Tukey HSD).

For the oDIDA treatments, the assumption of normality was met for each treatment and the control group at a 0.05 significance level (*p*-value >0.05). Homogeneity of variances was also confirmed (Levene’s *W* = 0.1801, *p* = 0.9687 > 0.05). The ANOVA results revealed statistically significant differences between treatments and the control (df = 5, *F* = 5549.92, *p* = 4.7013 × 10^–65^ < 0.05), with a partial eta squared value of 0.9982, indicating that 99.8273% of the variance in cell viability is explained by oDIDA concentration. The Dunnett post hoc test showed statistically significant differences between each treatment and the control (*p*-value< 0.05). Likewise, the Tukey HSD test showed significant differences among all treatment levels (*p* = 2.664535 × 10^–15^ < 0.05). The effect size was also quantified using Hedges’ *g*, with absolute values greater than 1 across all comparisons (see Figure 11), indicating a strong treatment effect.

**Figure 11:**
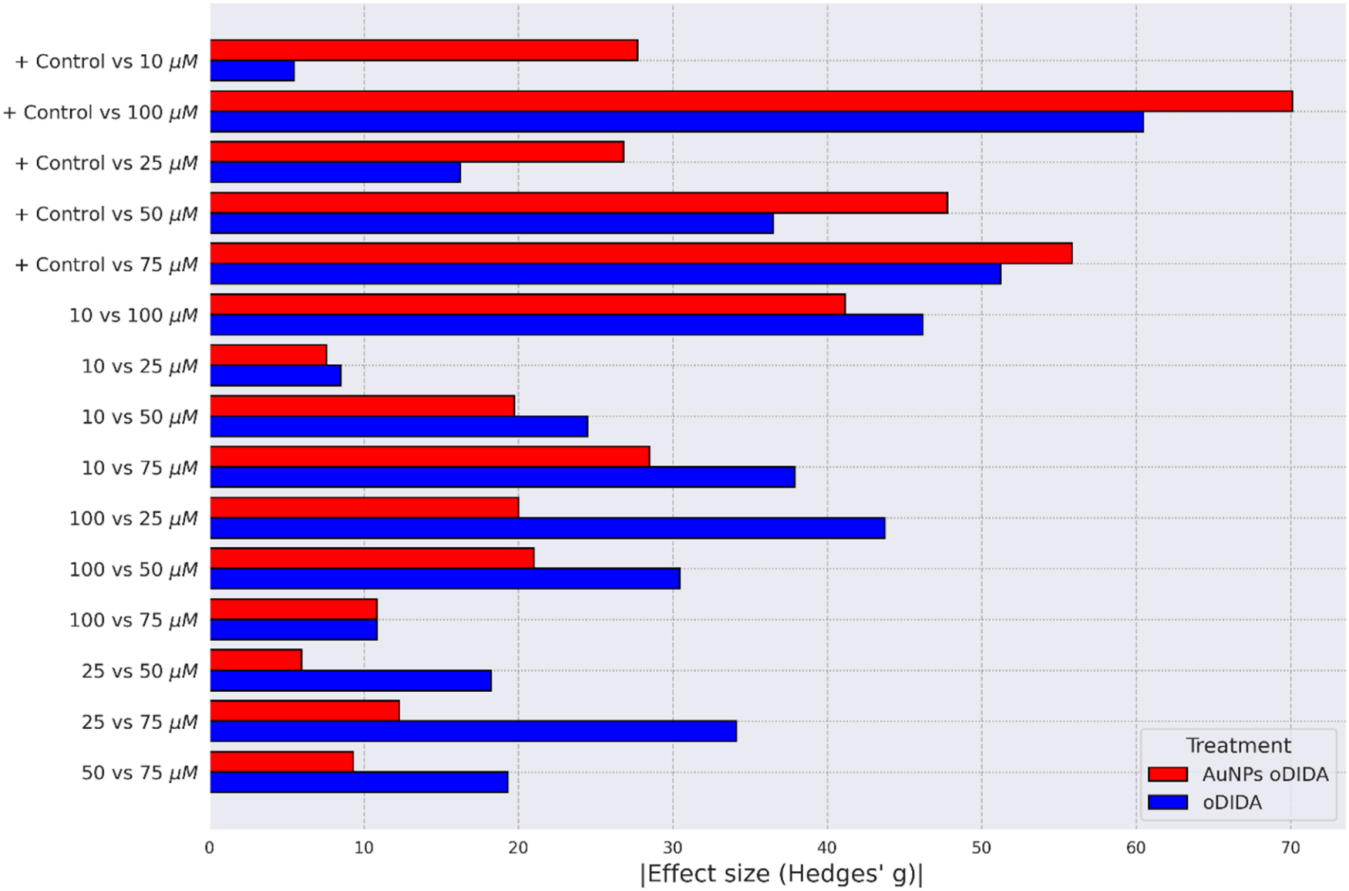
Effect size quantified by Hedges’ *g* coefficient for each comparison between treatments and the control in the XTT assay for the MDA-MB-231 cell line.

In the case of the AuNPs, normality was also assessed for each group. All but the 25 μM treatment satisfied the normality assumption at a 0.05 significance level (*p* = 0.034838 < 0.05 for the 25 μM group). Homogeneity of variances was confirmed (Levene’s *W* = 0.9880, *p* = 0.4349 > 0.05). The ANOVA results again revealed statistically significant differences among treatments and the control (df = 5, *F* = 4192.98, *p* = 3.8760 × 10^–62^ < 0.05), with a partial eta squared of 0.997716, indicating that nearly all the variability in cell viability is attributable to changes in AuNP concentration. Due to the violation of the normality assumption for one treatment, the ANOVA results were validated using the nonparametric Kruskal–Wallis test, which also confirmed significant differences (df = 5, *H* = 51.561177, *p* = 6.636681 × 10^–10^ < 0.05).

The Dunnett test revealed statistically significant differences between each treatment and the control group (*p* = 0 < 0.05). Similarly, the Tukey HSD test showed significant differences among all treatment means (*p* = 2.664535 × 10^–15^ < 0.05). These results were corroborated by the Hedges’ g effect size, which yielded values greater than 1 for all pairwise comparisons (see Figure 11).

In summary, the statistical analysis confirms that both oDIDA and oDIDA-reduced AuNPs significantly affect the cell viability. However, as shown in Figure 11, the effect size quantified by Hedges’ *g* is larger for the AuNPs compared to oDIDA alone when contrasted with the control, suggesting a higher cytotoxic activity for the AuNPs—potentially due to a synergistic effect. It is important to note that these effects were observed specifically in the MDA-MB-231 breast cancer cell line, highlighting the relevance of these findings in the context of targeted cytotoxicity.

For the MCF-7 cell line, the effect of oDIDA and the gold nanoparticles (AuNPs) synthesized using this compound on the cell viability was also investigated. The boxplot in Figure 12 illustrates a reduction in cell viability across all tested concentrations for both oDIDA and the AuNPs, compared to the control group. Notably, the decrease was more pronounced in the case of the AuNPs. To assess the statistical significance of these observations, the same statistical methodology previously applied to the MDA-MB-231 cell line was employed.

**Figure 12:**
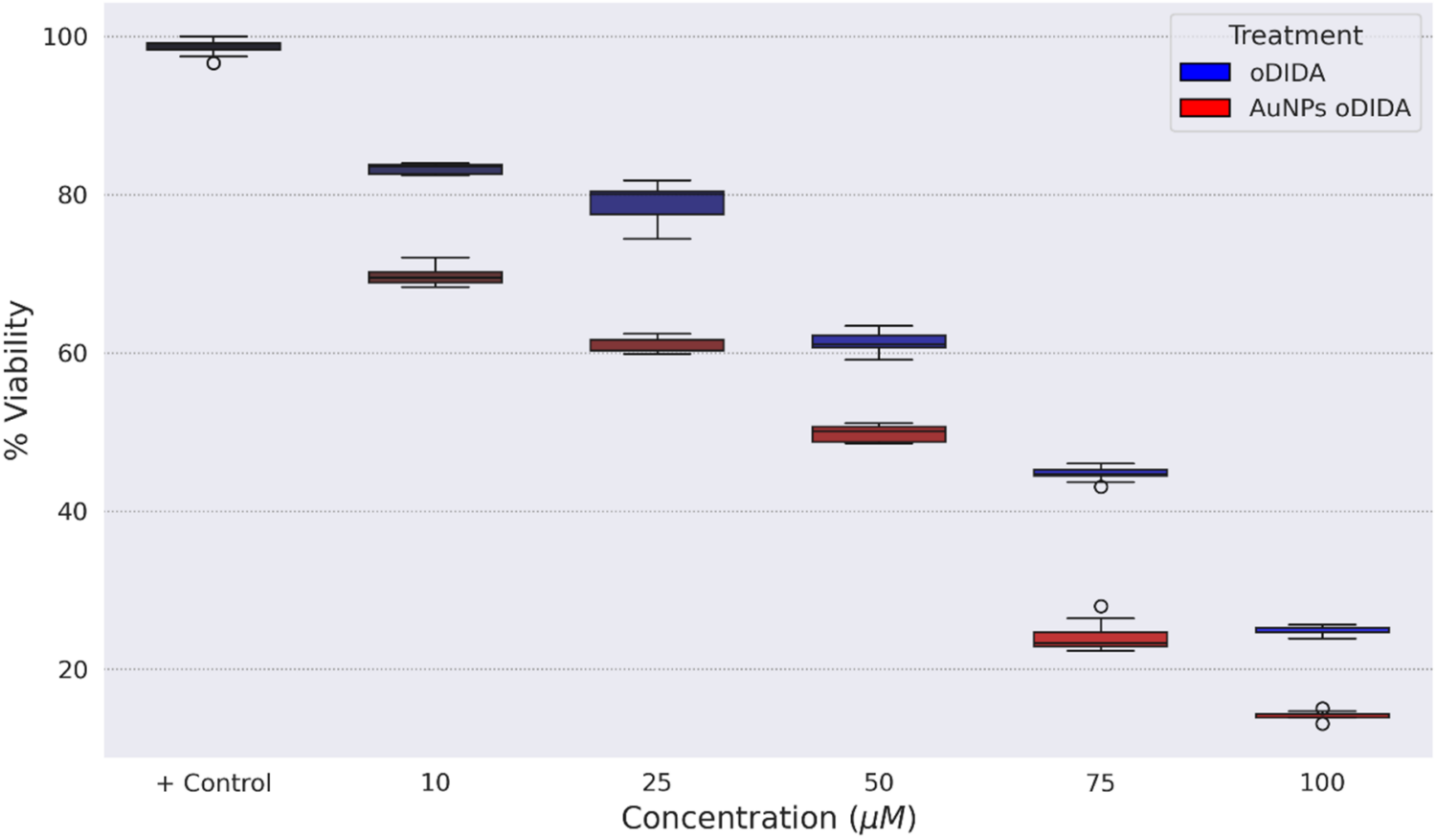
Boxplot of the cell viability percentage of the MCF-7 cell line for each treatment.

For all oDIDA treatments, the assumption of normality was satisfied for each concentration and for the control group (*p*-value >0.05). In addition, the assumption of homoscedasticity was confirmed (W-Levene = 2.075812, *p* = 0.084774 > 0.05). ANOVA results revealed statistically significant differences among the treatment groups and the control (df = 5, *F* = 3527.230591, *p* = 2.430918 × 10^–60^ < 0.05), with a large effect size as indicated by the partial eta-squared coefficient (η^2^ = 0.997286). This value indicates that a high proportion of the variability in cell viability is explained by changes in oDIDA concentration. Dunnett’s test revealed significant differences between the control and each treatment group (*p* = 0.0 < 0.05). Similarly, Tukey’s HSD test indicated statistically significant differences across all pairwise treatment comparisons (*p* = 2.664535 × 10^–15^ < 0.05). Effect size analysis using Hedges’ *g* yielded absolute values greater than 1 for all comparisons, supporting the conclusion of strong cytotoxic effects (see Figure 13).

**Figure 13:**
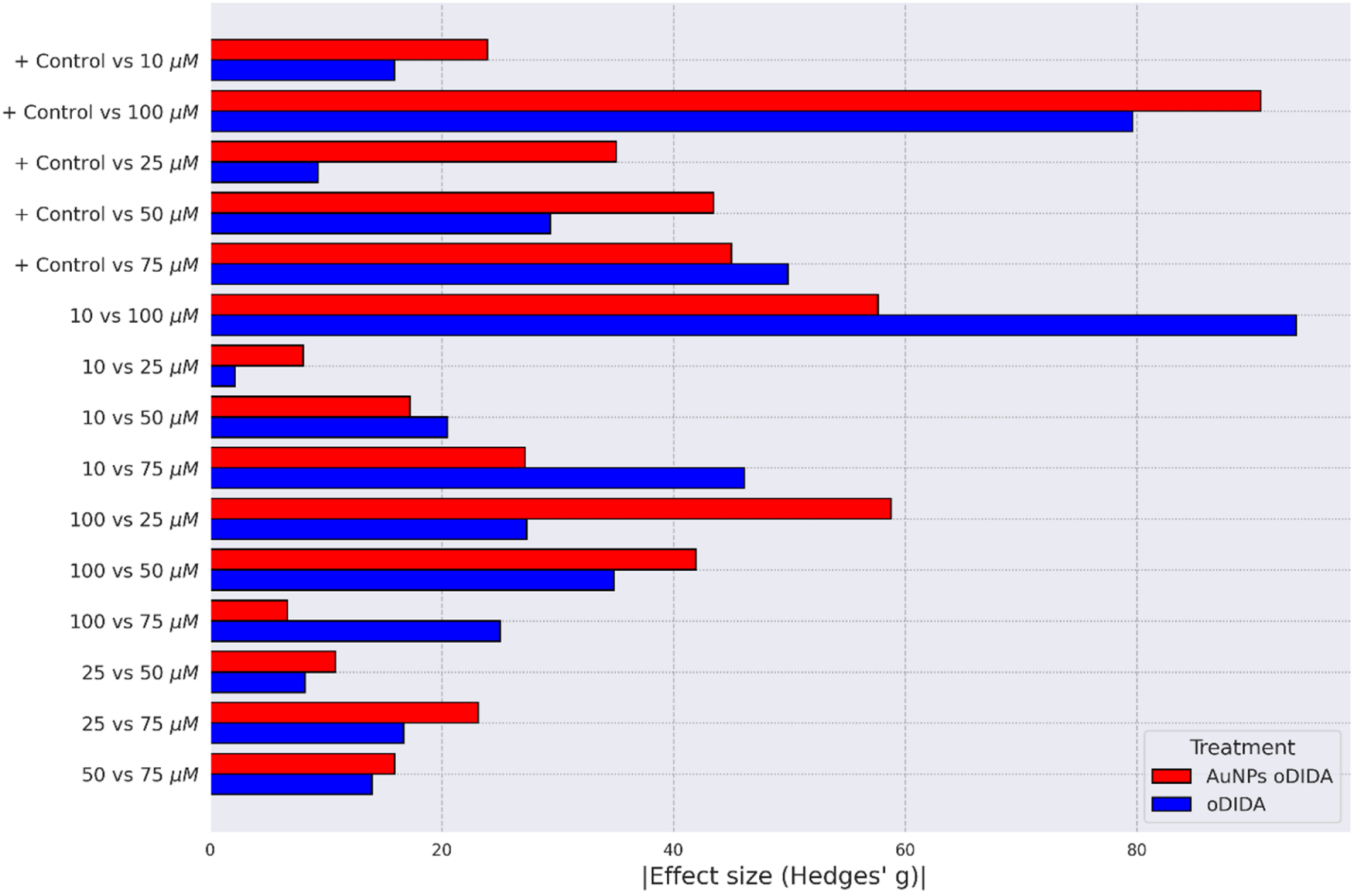
Effect size quantified by Hedges’ *g* coefficient for each comparison between treatments and the control in the XTT assay for the MCF-7 cell line.

To evaluate the statistical significance of differences between AuNP treatments, the same analytical approach was repeated. The assumption of normality was met for all groups except the 25 μM treatment (*p* = 0.039729 < 0.05). Homogeneity of variance was confirmed (W-Levene = 1.264506, *p* = 0.294629 > 0.05). ANOVA results indicated statistically significant differences between treatment and control means (df = 5, *F* = 6053.748469, *p* = 5.863010 × 10^–66^ < 0.05), with a very high effect size (partial η^2^ = 0.998417), suggesting that the observed reduction in cell viability is largely attributable to AuNP concentration. Due to the non-normality of one treatment group, the ANOVA findings were corroborated using the Kruskal–Wallis test, which also indicated statistically significant differences (df = 5, *H* = 51.592652, *p* = 6.538811 × 10^–10^ < 0.05). Subsequent Dunnett’s test comparisons showed significant differences between each treatment and the control (*p* = 0.0 < 0.05), while Tukey’s HSD confirmed statistically significant differences between all treatment groups (*p* = 2.664535 × 10^–15^ < 0.05). These findings were further supported by Hedges’ *g*, with all effect sizes exceeding 1 in absolute value (see Figure 13).

The overall analysis confirms that as observed with the MDA-MB-231 cell line, both oDIDA and the AuNPs exert cytotoxic effects on the MCF-7 cell line. However, Figure 13 shows that Hedges’ *g* values are consistently greater for the AuNP treatments than for oDIDA, indicating a stronger cytotoxic effect—except in the comparison between the control and the 75 μM treatment, where oDIDA exhibited a higher effect size. Nevertheless, the general trend supports the conclusion that AuNPs demonstrate a greater cytotoxic impact.

Finally, Table 5 shows the IC_50_ (mean inhibitory concentrations) obtained using the Hill equation and a nonlinear fit for oDIDA and AuNPs in both cell lines; these concentrations were tested in the MCF-10A line (Table 5). It can be seen that in the case of oDIDA, similar concentrations are presented; therefore, only the highest concentration (65.74 ± 1.06) was used for the experiment. The same applies to the case of AuNPs, and this could be due to the amount of AuNPs relative to their electrolyte present in each test; the ratio was 166 μM vs 833 μM.

**Table 5:** Cell Viability of MCF-10A from the IC_50_ Obtained from the Compounds Tested in MDA-MB-231 and MCF-7

cell type	compound	IC_50_ (μM)	%V MCF-10A
MDA-MB-231	oDIDA	65.74 ± 1.06 (*R* ^2^ = 0.9906)	95.13 ± 1.38
	AuNPs oDIDA	37.44 ± 1.52 (*R* ^2^ = 0.9812)	94.36 ± 2.14
	Cisplatin^a^	433.26	
MCF-7	oDIDA	65.24 ± 1.14 (*R* ^2^ = 0.9890)	95.64 ± 1.35
	AuNPs oDIDA	42.25 ± 1.67 (*R* ^2^ = 0.9753)	91.79 ± 2.12
	Cisplatin^b^	96.34 ± 0.81	

a Taken from reference (^62^).

b Taken from reference (^63^).

From the results shown in Table 5, it is possible to note that both oDIDA and its AuNPs generated a decrease in cell viability of the lines MDA-MB-231 and MCF-7 but, when in contact with the healthy epithelial line (MCF-10A) they did not present significant damage to these, therefore, both the electrolyte and the nanoparticles could work in future treatments against breast cancer.

Likewise, when compared to values reported in the literature for cisplatin, a widely used chemotherapeutic agent for the treatment of various cancers,^62,63^ both the oDIDA-based electrolyte and the corresponding gold nanoparticles (AuNPs) demonstrated a significantly lower average inhibitory concentration (IC_50_). This suggests a higher cytotoxic potency of the newly developed systems, potentially enabling effective treatment at lower doses. Moreover, cisplatin’s molecular structure incorporates a platinum atom, a rare and expensive metal, which contributes substantially to the overall cost of cancer treatments involving this drug. In contrast, oDIDA is composed exclusively of nonmetallic elements (carbon, hydrogen, nitrogen, and oxygen), which not only reduce material costs but also simplify synthesis and scale-up. These features make oDIDA-based systems a more economically viable and accessible alternative for anticancer therapy, particularly in low- and middle-income countries, where affordability remains a critical factor.

#### 3.3.1 Pharmacology ADME

Finally, the physicochemical properties of the studied compounds were predicted using the *SwissADME* online tool,^64,65^ which evaluates parameters relevant to drug-likeness and oral bioavailability. This platform specifically assesses six key molecular descriptors: lipophilicity, size, polarity, solubility, saturation, and flexibility (Figure 14). The analysis revealed that the molecule exhibits a high degree of unsaturation, primarily due to the presence of multiple aromatic rings within its chemical structure, which contributes to its rigidity and potential for π–π stacking interactions. In terms of polarity, the compound displayed values exceeding the conventional range, attributed to the presence of more than 10 polar atoms (mainly oxygen and nitrogen), which surpasses the threshold defined by Lipinski’s rule for optimal oral bioavailability. Despite this elevated polarity, the molecular size remains within the acceptable range for drug-like compounds, suggesting that the overall volume and molecular weight are not limiting factors for potential pharmacological application.

**Figure 14:**
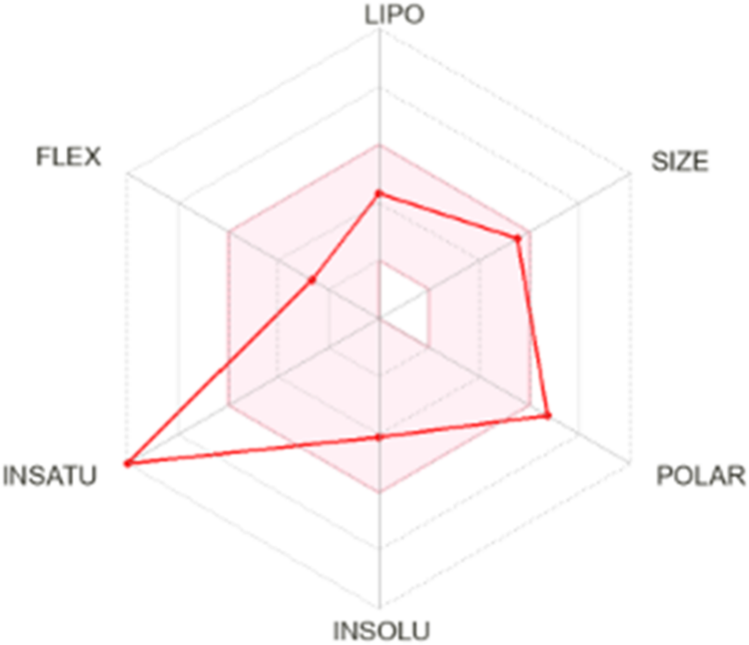
Radar chart showing the six predicted physicochemical properties of oDIDA.

Additional predicted physicochemical properties are summarized in Table 6. Notably, the macromolecule is not capable of crossing the blood–brain barrier (BBB), which reduces the risk of central nervous system (CNS) side effects and is advantageous for peripheral therapeutic applications. However, a significant drawback is its low gastrointestinal (GI) absorption, primarily attributed to its high topological polar surface area (TPSA), which exceeds 140 Å^2^. This elevated TPSA is a consequence of the molecule’s polarity, specifically the presence of 10 polar atoms (2 nitrogen and 8 oxygen atoms). Due to this limited absorption, the compound is not suitable for oral administration in its current electrolyte form and may not be effectively delivered through standard intravenous routes either.

**Table 6:** Predicted Pharmacokinetic Profile for oDIDA

TPSA^a^	149.36	ICYP^e^	No
GIA^b^	Low	PGP^f^	No
Sol^c^	0.0372	BBB^g^	No
Log *K*p^d^	–7.63	LP^h^	0

a Topological polar surface area (Å^2^),

b Gastrointestinal absorption,

c Solubility (mg/mL),

d Skin permeability (cm/s),

e CYP inhibitor,

f P-glycoprotein,

g Permeability of the blood-brain barrier,

h Lipinski.

The predicted aqueous solubility of 0.0372 mg·mL^–1^ indicates a poor water solubility, which can further compromise oral bioavailability. Additionally, the calculated skin permeability log *K*_p_ suggests low transdermal permeability, reinforcing the need for alternative administration strategies. On the metabolic front, the compound does not inhibit cytochrome P450 enzymes, indicating a low risk of metabolic drug–drug interactions. Similarly, the compound is not a substrate for P-glycoprotein (PGP), suggesting that it is unlikely to be actively effluxed from cells by this transporter, which simplifies its pharmacokinetics and clearance.

Finally, Figure 15 presents the “BOILED-Egg” model, a graphical bioavailability predictor. In this diagram, the absence of the compound from the yolk region confirms that it does not cross the blood–brain barrier, while its absence from the white region indicates low gastrointestinal absorption. However, its proximity to the GI absorption zone suggests that with structural optimization or appropriate formulation strategies, the molecule may be amenable to oral delivery.

**Figure 15:**
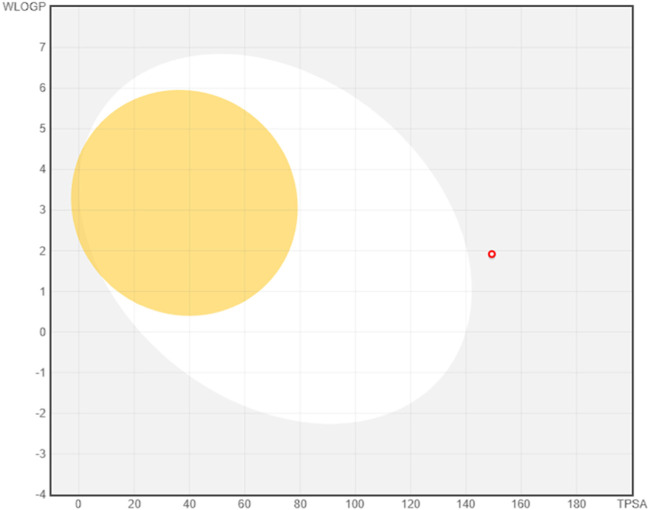
Boiled-Egg diagram for oDIDA.

## 4 Conclusions

oDIDA was used as a stabilizing and reducing agent for the green synthesis of gold nanoparticles. The quasi-spherical shapes of approximately 5.0 nm with a polydispersity of ± 2.5 nm were used to determine the cytotoxic effect on MCF-7 and MDA-MB-231 breast cancer cells. The average inhibitory concentration was decreased with AuNPs (37.44 ± 1.52) compared to the electrolyte (65.24 ± 1.14). Both IC_50_ were compared with cisplatin, a commercial drug that has been used for these cell lines previously, and it was found that the compound and its nanoparticles have a lower IC_50_ than the existing one. Finally, when testing the MCF-10A line, it was observed that both the electrolyte and its AuNPs did not present an adverse effect on healthy cells. During the *in silico* and *in vitro* studies, it was demonstrated that this compound can act as an alternative to the treatment of breast cancer.

## Supplementary Material



## Data Availability

The code generated in Python through Google Collab to obtain the statistical part shown in this work, as well as the programmed Tauc method, can be found at the following link: https://github.com/FDS116/Breast-Cancer-oDIDA.git.

## Notes

Table S1: Proteins for molecular docking; Table S2: Molecular dockings for oDIDA; Figure S1: Tauc’s method for AuNPs at 8 min; Figure S2: Tauc’s method for AuNPs at 35 days; Figure S3: Tauc’s method for AuNPs at 105 days; Table S3. Shapiro-Wilk test for oDIDA treatments (PDF)
