# Supplementary material for: Green Synthesis of Gold Nanoparticles and Their Cytotoxic Effect in Breast Cancer: An Experimental and Theoretical Study
Source: ACS Appl Bio Mater. 2025 Jul 31;8(8):7490–503. doi: 10.1021/acsabm.5c01172 (PMC12365886; doi:10.1021/acsabm.5c01172)
Supplement: Supplementary file 1 [file mt5c01172_si_001.pdf]

Green synthesis of gold nanoparticles and their cytotoxic effect in breast cancer, an experimental and theoretical study

Fausto Díaz-Sánchez[a], Jesús A. Arzola-Flores[a], Maura Cárdenas-García[b], Miguel A. García-Castro[a*], Juana D. Santamaría-Juárez[a]

[a] Facultad de Ingeniería Química de la Benemérita Universidad Autónoma de Puebla, 18 Sur y Av. San Claudio, C.P. 72570, Puebla Pue, México

[b] Laboratorio de Fisiología Celular, Facultad de Medicina de la Benemérita Universidad Autónoma de Puebla, C.P. 72570, Puebla Pue, Mexico

[*] Corresponding Author: miguel.garciacastro@correo.buap.mx

SUPPORTING INFORMATION

List of contents

Table S1: Proteins for molecular docking

Table S2: Molecular dockings for oDIDA

Figure S1: Tauc's method for AuNPs at 8 min

Figure S2: Tauc's method for AuNPs at 35 days

Figure S3: Tauc's method for AuNPs at 105 days

Table S3. Shapiro-Wilk test for oDIDA treatments

Table S1: Proteins for molecular docking

Protein	ID	Protein	ID	Protein	ID
IGF1R	2ZM3	MAPK1	2Y9Q	APEX1	7TC3
PARP1	6M3I	GSK3α	7SXF	ESR2	3OLL
PI3Kγ	3APC	CDK2	7VDU	FGF1	5EW8

Table S2: Molecular dockings for oDIDA

Complex	Docking	Interactions
PARP1-oDIDA	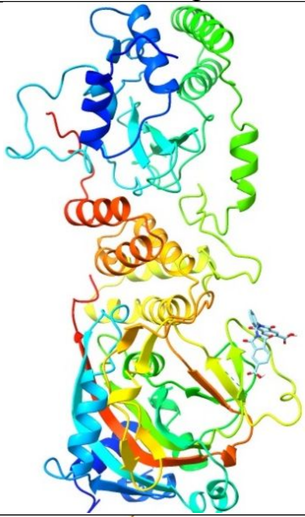	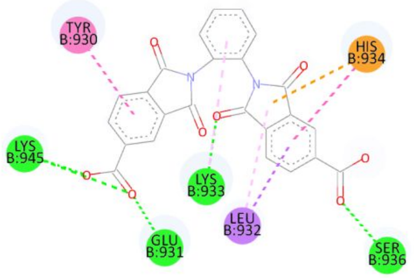
PI3Kγ-oDIDA	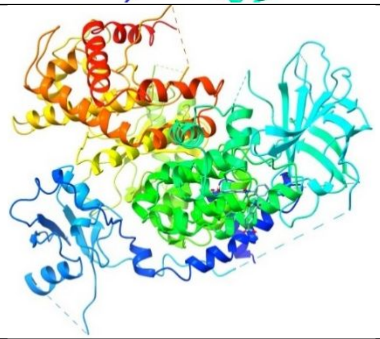	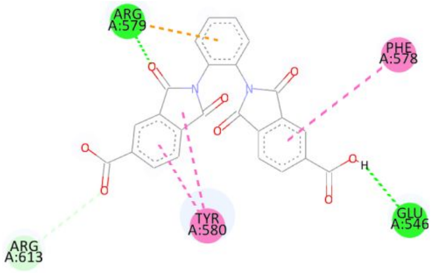
FGF1-oDIDA	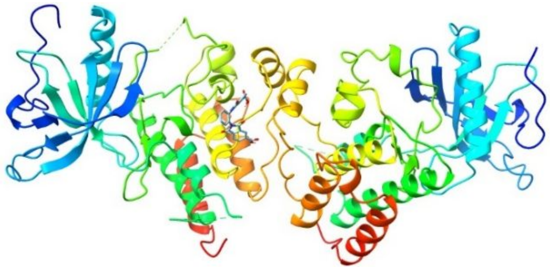	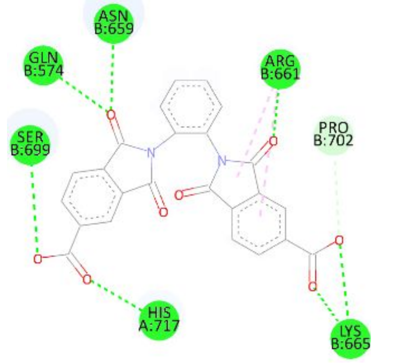

IGF1R-oDIDA

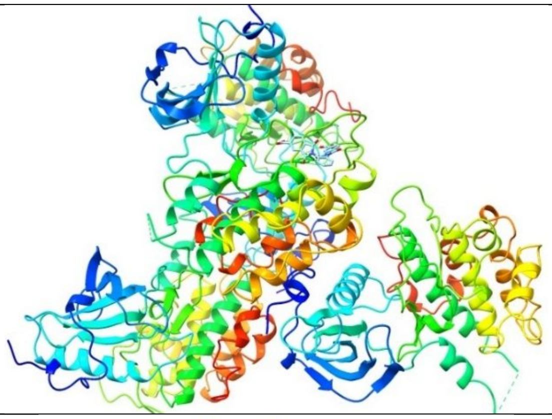

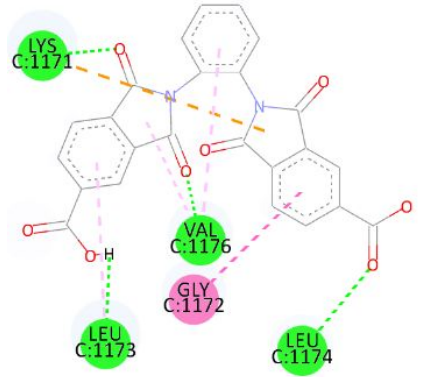

CDK2-oDIDA

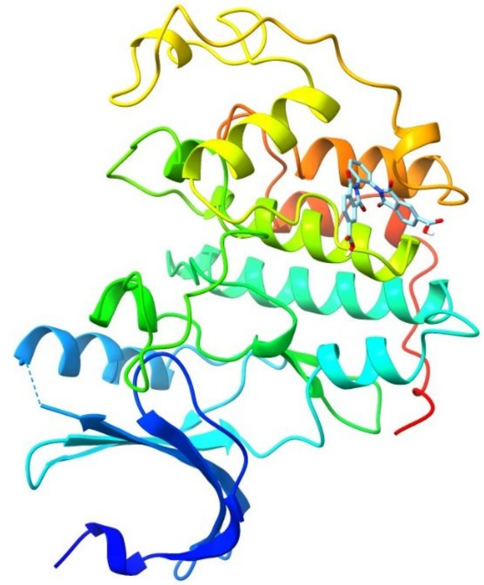

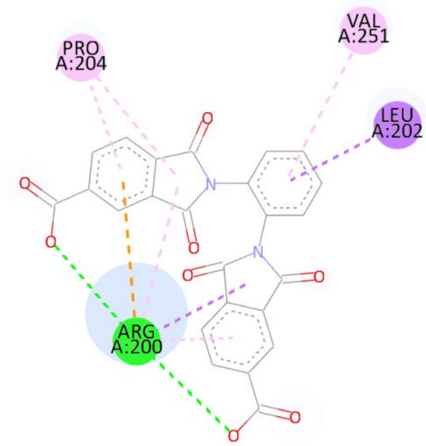

APEX1-oDIDA

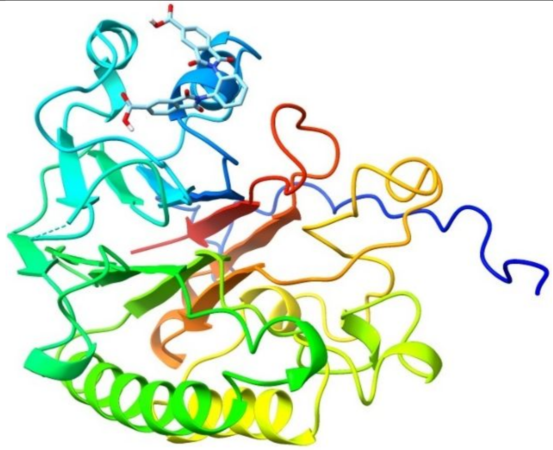

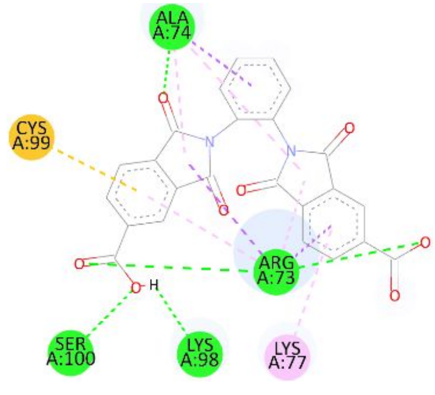

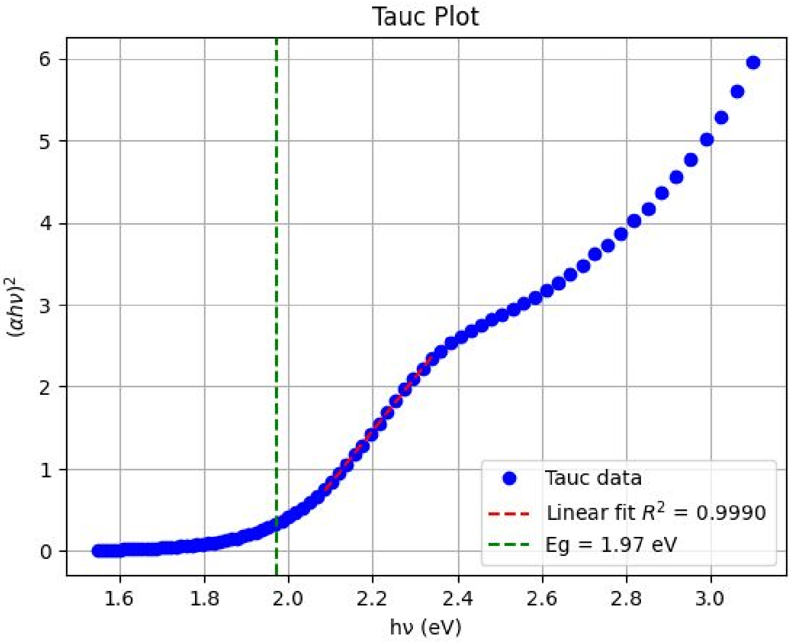

Figure S1: Tauc's method for AuNPs at 8 min

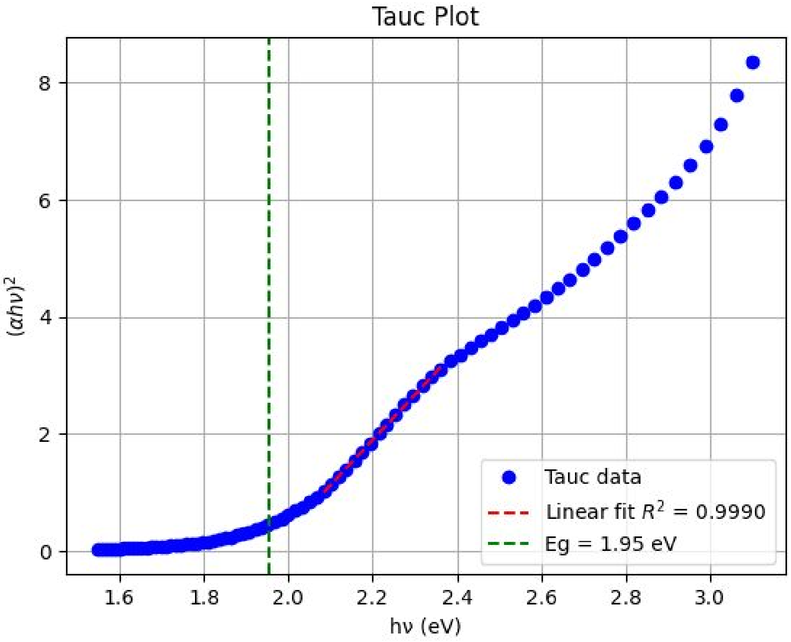

Figure S2: Tauc's method for AuNPs at 35 days

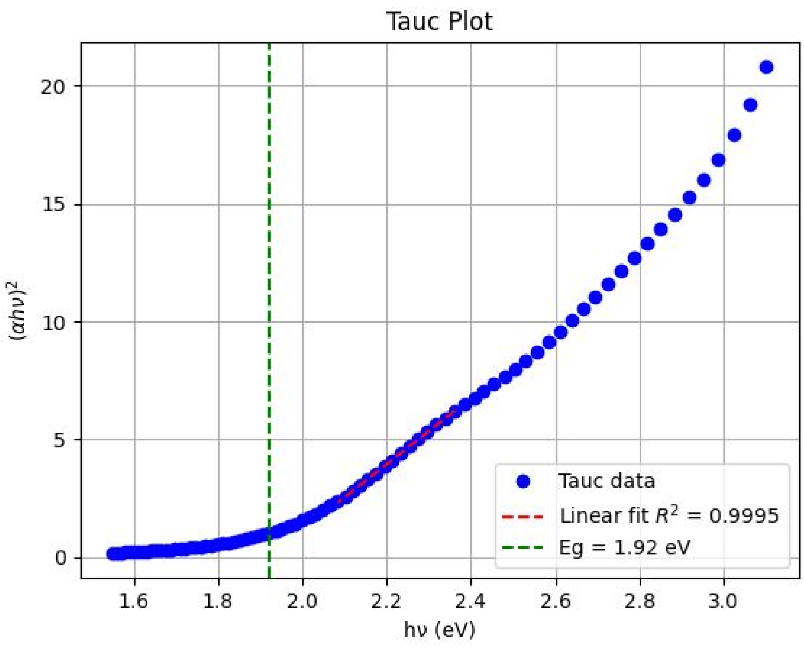

Figure S3: Tauc's method for AuNPs at 105 days

Table S3. Shapiro-Wilk test for oDIDA treatments

Group	W-Shapiro-Wilk	p-value
+ Control	0.9019	0.2636
10 µM	0.9232	0.4195
25 µM	0.9540	0.7348
50 µM	0.9440	0.6251
75 µM	0.9589	0.7876
100 µM	0.9238	0.4195
